# Guy's Hospital Reports. Second Series. No. VI

**Published:** 1846-01

**Authors:** 


Guy's Hospital Reports. Second Series. No. VI. October, 1845. Edited by Drs. *Barlow*, *Birkett*, and *Browne*, and Messrs. *Cock* and *Poland*. Octavo, from p. 173 to 324. Highley.

This number contains papers by Dr. Lever, Mr. Alfred Taylor, Mr. France, Dr. Nottingham of Liverpool, Mr. Bransby Cooper, Dr. Birkett, Mr. Gossett, Dr. Oldham, and Dr. Barlow.

### I. Two Cases of Labour, protracted by insuperable Rigidity of the Os Uteri. With Remarks. By *John C. W. Lever*, M.D.

Case 1. *Lingering First Labour; Sloughing of a Portion of the Mouth and Neck of the Womb; Delivery by Cephalotomy; Purulent Arthritis; Death.*—A young woman was taken in labour with her first child, on the 10th Sept. 1844. The pains were lingering till the morning of the 14th, when they became much more severe; the *os uteri* however was then not much larger than a shilling, and its edges were hard and rigid. She was bled from the arm, and the extract of Belladonna was applied freely to the mouth and neck of the womb. Dr. Lever saw her in the afternoon of the 14th, and recommended repeated doses of tartar emetic until complete nauseation was produced; this to be followed by a full dose of opium. No decided advantage was thereby gained. At a subsequent visit, next day, he found that, during a violent pain, a mass, consisting of a considerable portion of the cervix uteri in a most fetid state, had been expelled a short time previously. The child was extracted with the aid of the perforator and crotchet, " without greatly increasing the laceration that had occurred." Things went on tolerably well with the patient until the second day, when the right shoulder and elbow-joints became swollen and excessively painful; the metacarpal joint of the left index finger was also affected. There was no tenderness of the abdomen, and the lochia were free, but fetid. She appeared to improve for the next six days; but then (Sept. 22nd) " she was seized with shiverings, followed by intense heat and copious perspirations: almost every joint became swollen and painful, and her agony was extreme." She died on the morning of the 25th. No post-mortem examination was allowed.

" Many cases," says Dr. Lever, " are on record similar to the one I have related, in which, during labour, the os uteri has been torn completely off. The first specimen of the kind was produced before the Royal Medico-Chirurgical Society of London; and an account of that case, given by Mr. N. P. Scott, of Norwich, will be found in the 11th Vol. of the Transactions of that learned body. Soon after, a similar instance was reported by Steidele. In the 16th Vol. of the Dublin Journal will be found a brief report of two cases, related by Dr. Kennedy at the Meeting of the Dublin Obstetric Society. At page 52, of the same volume of the Dublin Journal, a case is related by Mr. Power, on the authority of his friend and colleague, Mr. Hugh Carmichael. The patient was a primipara, young, and unmarried." P. 179.

In reference to the arthritic symptoms, Dr. L. remarks that, of all the forms of puerperal disease, there is none so virulent and unmanageable as "puerperal rheumatism." The case is not unfrequently set down as rheumatic fever; but it is of a much more serious nature. Purulent infection of the system has taken place; and, in truth, we have one of the worst varieties of puerperal fever to deal with. Bouillaud has described several cases of the kind, under the appellation of "arthrite rheumatismale puerperale." It deserves notice that the urine was albuminous in the above case, when the arthritic affection made its appearance. Dr. L. is inclined to believe that these two phenomena are very frequently associated.

Case 2. *Lingering Labour—Section of the Os Uteri—speedy Recovery.*—A woman, at the age of 20, became affected with a *procidentia uteri* in consequence of lifting a heavy weight. A ball-and-socket pessary was ordered, at the time of the accident, to be worn. Two years subsequently, she was an in-patient of St. Thomas Hospital for the same displacement, which was then attended with so much inflammation as to require a protracted course of antiphlogistic treatment. When she got well, she married; 12 months after this date, she miscarried with a five-months' child. Four years since, she married her second husband; and, in March, 1844, became pregnant for the second time. When labour came on, the *os uteri* was found extremely rigid and undilatable. She was bled, purged, and nauseated with tartrate of antimony; but all with little effect. Although labour had continued for upwards of 48 hours, the uterine orifice merely admitted the tips of two fingers, and felt like a hardened ring. It was then thought advisable to divide "the whip-cord margin of the os uteri towards the posterior part of the sides of the pelvis, in the direction of either sacro-iliac synchondrosis. The incisions were made during the contraction of the uterus; the patient made no complaint; in fact, they gave her no pain." In the course of three hours from this time, the patient was delivered of a female child. Eventually she recovered.

In the remarks appended to this case, Dr. Lever very decidedly reprobates the practice of "artificial dilatation," as recommended by several writers, in the treatment of extreme rigidity of the uterine orifice: he professes himself "a strong advocate for the employment of incision." As a matter of course, the operation is, on the one hand, not to be had recourse to until the effects of bleeding, tartar-emetic, opium, warm baths, &c., have been fairly tried; and, on the other, it should not be delayed too long, until the patient's strength has become exhausted from protracted severe suffering. "The operation"—that of incising the *os uteri* in one or more places—"is unattended with danger, unaccompanied by pain, and, if rightly performed, free from copious or dangerous hæmorrhage." It does not appear that Dr. Lever has had occasion to perform the operation before the occurrence of the present case.

### II. Two Cases of Extra-Uterine Fœtation.

The other obstetric article in the present number is one by Dr. Oldham, the colleague of Dr. Lever at the hospital, in which an account is given of two cases of fatal extra-uterine pregnancy. In the first case, symptoms of Abortion came on during the fourth month of gestation; they subsided under the treatment employed. Ten days afterwards however, the patient, while in the water-closet—having previously over-exerted herself by walking—felt something give way within her; this feeling was followed by intense abdominal pain and syncope. She never rallied, but died on the following day. On *dissection*, it was found that the right Fallopian tube, which contained a fœtus of between three and four months' development, had become ruptured, and that, in consequence, a large quantity of blood had been effused into the abdominal cavity. As usual in cases of extra-uterine fœtation, the womb itself had become as much enlarged as if the fœtus had been contained within its cavity: its inner surface too was lined with a highly-developed deciduous membrane.

*Case* 2.—The woman considered herself about two months pregnant, when she was suddenly seized, while engaged in washing, with violent pain in the pubic region, followed by vomiting, syncope, and alarming prostration. She lingered for about 30 hours, and then expired. On *dissection*, four pints of blood were found extravasated into the abdominal cavity. On the left side of the fundus of the uterus, an aperture was seen, from which the blood had flowed. The uterus was considerably larger than in the unimpregnated state, and its inner surface was lined with "a rich and most exuberant growth of deciduous membrane." Close to the left horn of the uterus, a cavity had formed in the muscular substance of its parietes; this had burst outwardly. The excavation was small, and would hardly enclose a small horse-chesnut. No communication could be traced between it, and either the Fallopian tube or the cavity of the uterus. Although no trace of ovum could be discovered, yet the phenomena of the case led Dr. Oldham to the decided opinion that it was one of "interstitial or parietal extra-uterine pregnancy." Mr. Wharton Jones, who examined the preparation along with him, came to the same conclusion.

The observations, with which Dr. Oldham closes his narrative of these two cases, are at all events ingenious, if not quite satisfactory.

"The causes which have been assigned for misplaced gestation are very numerous and various; but, in my opinion, the two cases which have been related indicate the particular cause which is most frequently in operation:—I refer to the existence of false membranes, which, either by girthing the tube itself, or by fixing different parts of it, form a partial or complete stricture of its canal, or embarrass and restrain its movements, impeding it in its office as a conducting tube for the ovum. The development of the ovum without the womb is due, therefore, in these instances, to a mechanical error. It must have struck every one, who is in the habit of making post-mortem examinations, how frequently these membranes are found about the internal organs of generation. Sometimes they are so abundant, and so completely envelop the uterus and its appendages, that sterility is the necessary result. This is the case generally with women who have led abandoned lives, and is evidently caused from too-frequent sexual excitement. But very often they leave the passage of the tube open enough to permit the transmission of the semen; and the evil consequences are felt only either during or immediately after the escape of the ovum. A most important practical point to be noticed is, that the peritoneal inflammation which attends their formation produces but few symptoms compared with peritonitis in other parts of the abdomen. I have frequently found these false membranes in women whose history has not indicated any known attack of peritonitis; and I feel sure that they are produced in the course of illnesses, where the pain about the pelvic region is not a prominent feature. In the first case, the false membranes were no doubt formed about the tubes and ovaries after the first labour: they were not sufficient completely to obliterate the passage of the tubes, or to spoil the relation of their fimbriated extremities to the ovaries; but their presence diminished the chance of pregnancy, and caused, I suspect, the seven years' sterility which followed the first labour. I have known cases where, a few days after marriage, there has been pain and uneasiness on one or both sides of the pelvis, which has effectually been relieved by aperients, with hot narcotic fomentations and opiates; but sterility has followed; and I believe it to have been produced by the formation of these membranes around and about the uterine appendages. It is a matter of importance to bear this in mind, in reference to that class of puerperal diseases which are likely to produce them; and I have been in the habit of watching carefully those lingering traces of inflammatory action about the pelvis, which so often follow tedious labours or abortions, with the direct object of preventing them. The occasional use of leeches, and the application of the liniment. hydrarg. over the lower abdomen, or the empl. ammon. c̄ hydrarg., with strict adherence to rest, and a careful diet, will do much to effect this purpose." P. 278.

III. The case of Poisoning with Arsenic, detailed by Mr. Alfred Taylor, need not detain us; as there is nothing novel or unusual in its details, and no doubt could exist as to the agent employed by the criminal—who was tried at the Berkshire Lent Assizes, 1845, for the murder of his child, found guilty, and executed. Our only motive in making any allusion to it is to express our utter surprise and indignation at the scandalous facility with which the most destructive poisons, in large quantities too, can be procured by the lower classes of society. Is not the disclosure of the fact recorded in the following paragraph utterly disgraceful in a country like our own?

"Two bottles of white powder were produced by witnesses at the trial, to whom the prisoner had given them after the death of his children. One of these bottles was pronounced by the witness Carter to be similar to the one out of which she had seen the prisoner take the white powder to give to a neighbour.

"It was then necessary to determine the nature of the contents of each bottle, and for this purpose an analysis was required to be made during the trial. Each was found to contain white *arsenic* in fine powder, unmixed with any other ingredient, and the quantity in the two bottles, could not have been less than *five ounces.* Another bottle of a brown powder was also traced to the possession of the prisoner: this was proved to be *nux vomica,* which he had been in the habit of using for poisoning crows?" P. 196.

Ought such a state of things to be allowed to remain?

### IV. On the Pathology and Treatment of Fracture of the Neck of the Thigh-Bone. By *Bransby Cooper,* F.R.S.

Mr. Cooper, it is well known, adopts the opinion which was so much insisted upon by his celebrated uncle, and is very generally adopted by

English surgeons, that Fracture of the neck of the thigh-bone within the capsular ligament, if accompanied with separation of the two portions, is never healed by genuine ossific union. If this view be adopted, it follows that the patient will not be subjected to the protracted confinement in the horizontal position, which must as a matter of course be deemed necessary by those who take up the opposite opinion. The various sorts of apparatus, that have been contrived with a view of maintaining a permanent extension of the limb, are pronounced by Mr. Cooper to be "useless, because they are inapplicable to the purpose intended; and highly injurious to the patient, who, at the period of life at which this accident occurs, is ill capable of supporting protracted confinement in bed, and the infliction of continued extension and pressure of the limb." It is generally known that the neck of the thigh-bone begins to undergo, or perhaps has already undergone, very material changes in its physical character and configuration, when a person passes his fiftieth year or so.

"Let any one examine the upper extremity of a healthy adult femur, and the neck will be found branching from the shaft of the bone at an angle of 45°; but if an old bone be the object of observation, the head will be found depressed upon a level with the trochanter major, so that the neck forms a right angle with the shaft, and is infinitely shorter than it had been in youth, indicating an altered state of nutrition depending on the relative condition of epiphyses in old age and youth." P. 216.

Mr. C. then points out the various peculiarities of a bone, surrounded with a synovial ligament, that render it incapable of genuine ossific union. He particularly insists upon the absence of those very tissues, which unquestionably play an important part in the reparation (the early stage, at least) of the breach, when this occurs in the shaft or more solid part of the osseous structure. It is quite obvious that there cannot be the formation of a provisional callus all round the fractured ends of the femoral cervix—as invariably occurs in all fractures of the shaft; otherwise the acetabulum would be completely filled up, and all movement of the joint would be rendered impracticable. Those cases where the fracture exists partly within, and in part without, the capsular ligament, afford also a most convincing argument in favour of this view of the question; for then "the bony union takes place up to the very edge of the attachment of the synovial membrane, and no further."

So far, all is intelligible, and will meet with very general assent from British surgeons. But Mr. Cooper proceeds to advance another position, which seems to be rather more questionable; it is this:—"the necks of old femora . . . . . . . . have lost their power of generating phosphate of lime, *the deficiency of which it is that renders them so susceptible of fracture from the slightest violence.*"—(The Italics are ours). This somewhat startling assertion is alleged to be based upon the results of certain chemical examinations of the neck and the shaft of the femur, which Mr. Cooper undertook with the view of determining the relative proportion of the earthy materials in each, and "of ascertaining whether any change, or, if any, what change, took place in the composition of the neck of the thigh-bone in old age, which might prove sufficient to account for its tendency to bend and to break at this period of life."

"The specimens of bone were selected with care, and portions being sawn off, were weighed, and then burnt for a sufficient time in a muffle to destroy the whole of the animal matter, after which they were again weighed, and the result recorded. The quantity of bone burnt in each instance varied from 150 to 300 grains.

"The relative proportions of the phosphate and carbonate of lime were ascertained in a sufficient number of instances to prove that no perceptible difference worthy of remark occurred so as to mark, or even lead to the supposition that any change in the earthy constituents induced the physical alteration in the bone." P. 321.

Now the chief result (certainly unexpected) of these experiments has been to shew, that the osseous matter of the cervix femoris always contains a less amount of earthy materials than that of the shaft of the same bone,* and that this deficiency or disproportion is much more considerable in advanced than in middle life. In one case, in which the cervix had been fractured—the age of the patient was 68—100 grains of this portion of the bone were found to contain not more than 16 per cent. (about the usual average in bones affected with *mollities*), while the same quantity of the shaft contained as much as 53 per cent. of earthy matter.

"Aware," says Mr. Cooper, "that these results differ entirely from the received opinion that the liability to fracture in old age arises from an excess of bone earth, I have not hastily arrived at an opposite conclusion; nor should I have trusted to actual experiment as the basis upon which to found an argument militating so strongly against an established aphorism; but, reflecting on the mode in which the reparation of bone takes place in ordinary cases of fracture, it appeared to me little short of ridiculous to expect, in old age, an excess of bony matter, in a part which is so imperfectly vitalized, and which indeed, in common with all the epiphyses, is so many years consolidating into bone. In common fractures we find that animal matter is abundantly secreted within a few hours of the accident, whereas many days elapse before the formation of bone is even attempted; thus proving that a greater effort is necessary on the part of Nature to accomplish the production of bone, than is required in the reparation of any of the softer structures. When, therefore, the powers of the constitution are gradually diminished by age, it seems but reasonable to expect that a certain state of the system must ultimately arise, in which bone earth is liable to be deposited in the more organized and vascular parts of the body; and hence, consequently, arises a diminution of the solid constituents of the bones, and especially in those which possess the least degree of vitality. The epiphyses are the first parts of the osseous system which become deteriorated from the want of earthy deposit, and the deficiency is made up by the deposition of cartilage, gelatine, and other substances more easily secreted than bone; whilst the tendency, at this period of life, occurs to the secretion of bone in the more highly-organized structures, as in the aorta, lungs, prostate gland, and kidneys, and not unfrequently giving rise to ossifications, or the formation of phosphatic calculi in the bladder or prostate gland. In support of this opinion we find that the complaints alluded to for the most part occur at that period of life in which we are led to expect a want of power in the imperfectly-vitalized structures of the body; and these experiments may serve to convince us that we ought not to expect bony union in such situations, and that this becomes all but impossible after the middle period of life, as there ensues then a disposition rather to diminish than increase the due pro-

---

* If Mr. Cooper's experiments be correct, the statement, here made respecting the neck of the thigh-bone, is believed to be equally true of the epiphyses of long bones in general.

portion of solid matter, and most especially in the articular extremities of the bone." P. 224.

There is more than one physiological and pathological position in this extract which will doubtless somewhat discompose the reader, who has always been accustomed to believe that the bones in old age contain more earthy matter than they did in middle life. We need scarcely say that Mr. Cooper's chemical experiments must be repeated by those accustomed to such researches, before we can receive them as entitled to much authority; the discordance between the results obtained by him and other experimenters is so very great. According to his examinations, the average quantity of bone earth in the shaft of the femur in an old person does not exceed 55 per cent.; whereas it is usually stated as high as 75, and even considerably more. We wonder that Mr. Cooper did not think of testing the results of his calcining experiments, by ascertaining the amount of earthy matter lost by subjecting the portions of bone to muriatic acid. Had he done this, he would have been enabled, at the same time, to examine not only the quantity, but also the structure, of the cartilaginous substance left behind.

### V. Select Clinical Reports, with Observations. By *Geo. H. Barlow,* M.A. and M.D.

Dr. Barlow has shewn, in a previous paper (noticed in our number for January last), the influence of obstruction in different parts of the Bowels, and also of impediments to the free passage of the blood through the portal system of the Liver, upon the quantity of fluid secreted by the Kidneys.

In working out the proposition there enunciated—" If a sufficient quantity of water cannot be received into the small intestines; or if the circuit through the portal system into the vena cava ascendens, or thence through the lungs and heart into the systemic circulation be obstructed; or if there be extensive disorganization of the kidneys; the due secretion of urine cannot be effected"—he now proceeds to examine the effects of obstructed circulation through the Heart and Lungs upon this secretion.

The first case related is one of *Dropsical Effusion from Pulmonary Obstruction,* arising from neglected Bronchitis; the legs had become very œdematous, and there was a good deal of fluid in the abdomen. The bowels were purged with the compound jalap powder, and the kidneys stimulated with saline diuretics and nitric æther. A pill—consisting of one grain of Pil. Hydrarg., Pulv. Scillæ, and Pulv. Ipecac. and two grains of Extr. Hyosciami—was also given night and morning. In the course of ten days, the woman had lost her cough and dropsical swelling:—she was soon convalescent.

"The effects," says Dr. B. " upon other organs, of repeated or long-continued bronchitis, are, as is well known, distention of the right side of the heart, with general venous obstruction, engorgement of the liver, œdema of the lower extremities; and, in extreme cases, the obstruction to the circulation through the right side of the heart may be such as so produce œdema of the face and superior extremities; but this, I believe, rarely happens until the distention has been so great, and so long continued, as to lead to organic change." P. 286.

The next case is headed *Bronchitis with Emphysema of the Lungs—Ascites and Anasarca—mild antiphlogistic and diuretic treatment—Recovery.* The symptoms rapidly subsided under the use of purgatives and mild diuretics.

In both of these cases, the passage of the blood through the lungs being impeded, the due secretion of the urine was interrupted; for, in the first instance, the patient is stated to have passed hardly four ounces in twenty-four hours; and in the second, it was scanty. He found that, when this obstruction was diminished by the reduction of the bronchial inflammation, the further secretion of urine was readily effected. We have, then, in these cases, evidence of the truth of the proposition that, when the passage of the blood through the pulmonary vessels is obstructed, the due secretion of urine cannot be effected.

It will be obvious that in all such cases there must necessarily be congestion, in a greater or less degree, of the Liver; this congestion arising from the obstruction to the portal circulation, caused by the pressure backwards of the blood in the right cavities of the Heart. Hence the importance and efficacy of stimulating the hepatic function, and of causing a copious secretion from the mesenteric vessels by the use of hydragogue purgatives. The application too of blisters, over the part of the lungs chiefly affected, should seldom be omitted; and the atmosphere of the patient's room should always be kept warm and rather moist. This precaution is often overlooked; but it is one of much importance.

The next three cases recorded in Dr. Barlow's paper, are instances of "Dropsical Effusion connected with disease of the Heart." In the *first*, this disease was extreme dilatation of the right cavities, and also of the left auricle, with slight narrowing of the auriculo-ventricular valves; in the *second*, there was a flabby large heart, and a granular tuberculated state of the mitral valve; and in the *third*, there was dilatation and hypertrophe of both ventricles, and also disease of the aortic valves. Dr. Barlow thus explains the *modus operandi* of these various lesions in inducing the same morbid consequences:—

"The reason of the same effects having been produced by these different lesions is sufficiently obvious; namely, that the obstruction was propagated backwards through the whole course of the circulation; the left ventricle being unable to empty itself, the passage of the blood into it was of course impeded, and the result was the same as in Case 6, where there was an obstruction to the passage of the blood into the ventricle, and Case 7, where there was regurgitation from it; the series of diseased action being, obstruction to the emptying of the left ventricle, both by contraction of its aortic orifice, and imperfection of its valves—dilation of that ventricle, increasing the difficulty of its contraction more than was compensated for by the hypertrophy—accumulation of blood in that ventricle—engorgement of the auricle, then of the lungs—distention of the right heart, obstruction to the flow of blood through the cavæ—turgescence of the face, and congestion in the brain—engorgement of the liver, obstruction to the portal circulation and diminished secretion of urine—ascites and œdema." P. 303.

He then discusses some points in the history of valvular and other murmurs of the Heart ; but it is really unnecessary to follow him in this discussion, as it must be quite obvious to every dispassionate enquirer that we are far from having any stable or trustworthy signs to indicate, with any degree of certainty, the seat and nature of the valvular lesion that is present in any particular case. As to attaching much consequence to certain "systolic murmurs," as evidences of the regurgitation of the blood through this or that orifice of the heart, the attempt has hitherto met with but little success. One observer has generally managed to differ very materially in his speculations from his immediate predecessor ; and a third experimenter soon discovers that both his learned brethren have been at fault. Dr. Hope has given it as his opinion that "a decidedly jerking pulse denotes a free aortic regurgitation, and a decidedly weak and irregular pulse bespeaks great mitral contraction or free regurgitation." Dr. Barlow admits that this "proposition partially holds good in many, or even in most, cases;" but he then proceeds to observe "that considerable disease of this (mitral) valve may exist without a small or irregular pulse ; and that we may have the very feeble pulse spoken of, and the mitral valve perfectly healthy." He sums up his observations in the following Corollary.

"If the reasoning which has been adduced above be correct, it must follow that intermission of the pulse is more likely to be produced by an obstruction to the flow of the blood into the left ventricle, than by regurgitation through the auriculo-ventricular orifice; since, in the former case, there is a want of that supply of the blood whereby the ventricle is stimulated to contraction; and in the latter there is not *necessarily* any great deficiency in this respect, although the force and volume of the arterial pulse may be greatly diminished by a portion of the blood being thrown back into the auricle." P. 309.

The last case, that is related, is one of a rather singular nature; *the right kidney was obliterated, and the left one was converted into a large sac or cyst.* The patient's constitution was cachectic and broken down, partly from intemperance, and in part from long-continued tendency to ague. When admitted into the Hospital, his abdomen was large and flabby, with an internal swelling on the left side, occupying the lumbar, half the umbilical, and most of the left hypochondriac regions ; there was an incessant gnawing pain in the part. Dr. Barlow candidly confesses that he regarded this tumour as formed by an enlargement of the Spleen—a frequent sequela, it is well known, of protracted agues. The urine was abundant, pale, of very low specific gravity (1002), and neutral. The man gradually became weaker and weaker; symptoms of coma came on; and he died in an epileptic fit.

*Dissection.*—The tumour on the left side of the abdomen proved to be the left kidney, which had become converted into a large sac that contained not less than two pints of limpid urine; its cortical substance was "of a pale fawn colour, and soapy cartilaginous consistence, honeycombed by masses and deposits of cheesy purulent matter. In the left ureter was a constriction by inflammation, amounting to perfect obliteration at that point, about an inch from the pelvis of the kidney; the ureter above the stricture being enormously dilated, and below it about twice its natural size. The right kidney was completely obliterated, a very small mass of fleshy fat alone shewing where it had been: the supra-renal capsule being rather larger than usual: ureter very small, pervious."

A very curious circumstance in this case was, that, in spite of the extreme degeneration of the renal apparatus, the excretion of the urine—of a limpid character indeed—was abundant till within 36 hours of the patient's death: just before this event took place, a pint had been drawn off by means of the catheter.

"If we apply to this fact," says Dr. Barlow, "the modern theory of renal secretion (for which we are indebted to Mr. Bowman), namely, that the water is poured out from the little tufts which form the nuclei of the Malpighian bodies, whilst the solid ingredients are excreted from the lining membrane of the tubes which lead from these bodies, we shall be led to the belief, that, in the case before us, the seat of the disease was, primarily, in those tubes, whilst the Malpighian tufts remained, as far as their functions were concerned, intact. It is indeed rather difficult to understand how disease could have advanced so far in the tubes, and yet have allowed a free passage through them from the tufts; still the thing is conceivable, and we have almost demonstrative evidence that it was so." P. 314.

This valuable paper cannot fail to add to the reputation of its zealous and talented author.

VI. The remaining articles in this Number of these Reports do not require particular notice. Mr. France relates a curious case of Ossification and Dislocation of the Crystalline lens; and Dr. Nottingham one of Popliteal Aneurism, in which the femoral artery was tied with complete success. The patient, however, died within fourteen weeks after the operation from Delirium Tremens, brought on by the abuse of ardent spirits. An account is given of the appearances, found upon dissection, in the arteries of the affected limb.

The papers by Dr. Birkett and Mr. Gossett contain abstracts of the half-yearly reports, the one medical and the other surgical, of the Clinical Society attached to Guy's Hospital School. We may cull two or three short paragraphs, having a bearing upon some practical point.

*Treatment of Chorea.*—" In thirteen cases of chorea the sulphate of zinc was administered, and in twelve, with perfect success; three, in the persons of young chlorotic women, were treated with iron, two of whom were dismissed cured; and one case, of three months' standing, occurring just before puberty without assignable cause, yielded to the oxide of zinc, where the sulphate, electricity, and arsenic had been used in vain.

"In nine cases the disease was preceded by fright; and in two by rheumatic affection complicated with cardiac sounds." P. 233.

*Treatment of Lepra.*—"The very frequent occurrence of a disease of more inconvenience than danger, the obstinacy in resisting treatment, and the liability to recur, have called attention to lepra. The bath of pyroligneous acid has been found a very efficacious remedy, especially when combined with the internal administration of pitch. The ung. picis liq. has also been useful as an external application." P. 245.

*Fatality of Albuminuria.*—" The cases of albuminuria tell the usual tale: out of 15 cases five only were cured, and in four of these the complaint followed scarlatina. The remaining case referred to as cured occurred in the course of an attack of erysipelas of the face. The case relieved was a complication of bronchitis with cardiac disease; and the remedies were, cupping to the loins, with antimony, followed by the sesquichloride of iron. The complications are of a two-fold nature; 1st, those which either precede or accompany the disease in the early stages; 2dly, those which do not arise till near the close of life. Among the first may be classed scarlatina and the diseases of the circulating organs—chiefly with respect to the direct performance of their functions—which are generally of long standing at the period of attack; and among the latter, affections of the head, indicated by convulsions; of the chest, by bronchitis and pneumonia; and of the general system, by anasarca and a tendency to serous inflammation." P. 247.

The Medical School of Guy's Hospital is, at the present time, unquestionably one of the most efficient and best-managed institutions in any part of the kingdom. Its pupils will have to blame themselves, if they do not become able and accomplished practitioners in after-life; for they have great and manifold advantages in having such talented and zealous men to guide and instruct them in their studies.

